# Analysis of Plasma MicroRNAs as Predictors and Biomarkers of Aging and Frailty in Humans

**DOI:** 10.1155/2018/7671850

**Published:** 2018-07-18

**Authors:** Iryna Rusanova, María E. Diaz-Casado, Marisol Fernández-Ortiz, Paula Aranda-Martínez, Ana Guerra-Librero, Francisco J. García-García, Germaine Escames, Leocadio Mañas, Darío Acuña-Castroviejo

**Affiliations:** ^1^Departamento de Fisiología, Facultad de Medicina, Centro de Investigación Biomédica, Parque Tecnológico de Ciencias de la Salud, Universidad de Granada, Granada, Spain; ^2^CIBERfes, ibs.Granada, Granada, Spain; ^3^CIBERfes, División de Medicina Geriátrica, Hospital Virgen del Valle, Complejo Hospitalario de Toledo, Toledo, Spain; ^4^CIBERfes, Servicio de Geriatría, Hospital Universitario de Getafe, Madrid, Spain; ^5^UGC de Laboratorios Clínicos, Complejo Hospitalario de Granada, Granada, Spain

## Abstract

Although circulating microRNAs (miRNAs) can modulate gene expression and affect immune system response, little is known about their participation in age-associated frailty syndrome and sarcopenia. The aim of this study was to determine miRNAs as possible biomarkers of age and frailty and their correlation with oxidative and inflammatory state in human blood. Three inflammation-related miRNAs (miR-21, miR-146a, and miR-223) and one miRNA related with the control of melatonin synthesis (miR-483) were analyzed. Twenty-two healthy adults, 34 aged robust, and 40 aged fragile patients were selected for this study. The expression of plasma miRNAs was assessed by RT-qPCR; plasma cytokines (IL-6, IL-8, IL-10, and TNF*α*) were analyzed by commercial kits, and plasma advanced oxidation protein products (AOPP) and lipid oxidation (LPO) were spectrophotometrically measured. Fragile subjects had higher miR-21 levels than control subjects, whereas miR-223 and miR-483 levels increased at a similar extend in both aged groups. All cytokines measured increased in aged groups compared with controls, without differences between robust and fragile subjects. The fragile group had a TNF*α*/IL-10 ratio significantly higher than robust and control groups. Aged groups also had higher AOPP and LPO levels than controls. Women presented higher AOPP and LPO levels and increased expression of miR-483 compared with men. Positive correlations between miR-21 and AOPP and between miR-483 and IL-8 were detected. The expression of miR-21 and the TNF*α*/IL-10 ratio were correlated positively with the presence of frailty, which suggests that these markers can be considered as possible biomarkers for age-related frailty.

## 1. Introduction

One reason that aging is emerging as a key policy issue is that both the proportion and absolute number of older people in populations around the world are increasing dramatically. Aging is a global problem affecting both developed and developing countries. According with the World Health Organization report of 2015, the older adult population in sub-Saharan Africa is expected to grow faster than anywhere else, increasing from 46 million in 2015 to 157 million by 2050 [1]. In terms of life expectancy at birth, the EU is a world leader, and according to “*The 2015 Ageing Report*” by European Commission, the life expectancy at birth for males is expected to increase by 7.1 years from 2013 to 2060 period, reaching 84.8 in 2060 [2]. For females, it is projected to increase by 6.0 years, reaching 89.1 in 2060. As a result, the demographic old-age dependency ratio (people aged 65 or above relative to those aged 15–64) is projected to increase from 27.8% to 50.1% in the EU as a whole over the mentioned period. Similar changes are expected in many other countries.

Apart from the usual age-related diseases, progressive loss of muscle mass and function associated with aging limits the physical capabilities of the older population and increases the cost of health care by the public health system. This age-related muscle wasting has been termed sarcopenia. The preliminary concept of sarcopenia as an age-related loss of muscle mass and function [3] was updated in 2010 by the European Working Group on Sarcopenia in Older People (EWGSOP) focus on a generalized loss of muscle mass and strength with the risk of physical disability, poor quality of life, and death [4].

Sarcopenia is one of the manifestations of frailty, a clinical condition of aged adults that increases risk for poor health outcomes including falls, disabilities, hospitalization, and death [5]. Clinical features of frailty are related to chronic inflammation associated to the age (inflammaging), oxidative stress, mitochondrial dysfunction, insulin resistance, aging-related loss of anabolic hormones, diminished strength, and tolerance to physical activity [6]. The prevalence of frailty of adults aged 65 and older is 12% in the USA, increasing up 25% in the group of 85 years or older [7]. Similar data have been reported in other countries including the European Union [2].

Circulating noncoding RNAs (miRNAs) are small RNA molecules that, depending upon base pairing to mRNA, mediate mRNA cleavage, translational repression, or mRNA destabilization [8, 9]. miRNAs or miRs are involved in crucial cellular processes, and their deregulation has been described in different diseases, including aging, sarcopenia, and age-related musculoskeletal impairments [10–12]. Due to the role of some miRNAs to promote inflammation, changes in expression of miRNAs throughout life, and their easily measurement in blood, it is suggested that miRNAs may be candidates for an early diagnosis of frailty in the elderly.

Thus, as a potential noninvasive method to study frailty, we consider it worthwhile the determination of some miRNA levels in blood and analyze whether changes in circulating miRNAs correlate with oxidative stress and inflammation during aging and frailty. We determined miR-21, miR-146a, and miR-223, three miRNAs related with inflammation, and miR-483, which is related with the control of melatonin synthesis, the hormone with anti-aging properties [13].

## 2. Materials and Methods

### 2.1. Participants

Control samples were collected from healthy volunteers. Samples from frailty and nonfrailty aged groups of subjects were obtained from the cohort of the Toledo study for healthy aging, whose characterization was published elsewhere [14]. The participants in the study were classified into three groups: young healthy group (control), aged healthy group (robust), and aged fragile group (fragile) (Table 1). Informed consent was obtained from all subjects and from the Hospital Ethical Committee, according to the 1983 revised Helsinki Declaration of 1975. The final study was approved by the Andalusian Ethical Committee (ref. CEEA: 462-CEEA-2013). The diagnosis of frailty in aged population was based on the Fried criteria (weakness, low speed, low physical activity, exhaustion, and weight loss) [15]. As an indirect measurement of sarcopenia, a hand grip strength test was used [16].

### 2.2. Assessment of Frailty and Physical Performance

The Fried criteria used for diagnosis of frailty are described as follows [15]: weakness is defined as the worse quintile of maximum strength on the dominant hand adjusted for sex and body mass index (kg/m^2^), strength was measured with a Jaymar hydraulic dynamometer, according to the standards of the Hispanic EPESE, weight was measured with a SECA precision scale, and height was measured with a stadiometer on a wall without a skirting board [17]. Physical performance was assessed with the following criteria: (1) low energy, subjects were classified as having “low energy” when they provided a positive answer to any of the following two questions from the CES-D (Center for Epidemiologic Studies Depression Scale) [18]: “I felt that anything I did was a big effort” and “I felt that I could not keep on doing things” at least 3 to 4 days a week; (2) slowness, defined as the worse quintile in the three-meter walking speed test, adjusted for sex and height according to the standards of the Short Physical Performance Battery [19]; and (3) weight loss, defined as unintentional weight loss of 4.5 kg or more in the last year. One point was assigned to each variable and built a score as the sum of points for all of them. According to this score, aged subjects were classified as nonfrail or robust (0 points), prefrail (1-2 points), and fragile (≥3 points). For this study, we used samples of robust and fragile patients only. The data of physical activity of the patients were obtained with the Physical Activity Scale for the Elderly (PASE). This questionnaire was specifically developed to assess the physical activity in epidemiological studies in people ≥ 65 years [20]. Physical dependence was evaluated based on measures reflecting the personal persuasion of need of help for the accomplishment of activities of daily living. The information about the presence of hypertension was based on medical diagnosis.

### 2.3. Samples

Blood samples (10 mL) were collected from the antecubital vein between 8:00 and 9:00 a.m. after 8–10 hours of fasting. The samples were collected in vacutainer tubes with EDTA-K_2_. Blood was centrifuged at 1900 ×g for 10 min at 4°C, and plasma and erythrocytes were separated. Aliquots of plasma were transferred into RNase-free microcentrifuge tubes and stored at −80°C until the assays were performed. Once thawed, 250 *μ*L of all plasma samples was centrifuged at 16000 ×g at 4°C for 10 min to remove cryoprecipitates. 100 *μ*L of plasma supernatants was used for miRNA isolation.

### 2.4. Plasma miRNA Profiling and Data Analysis

Plasma miRNAs were extracted using the miRNeasy Serum/Plasma Kit (Qiagen, Werfen España, Barcelona, Spain) by following the manufacturer's instructions. Reverse transcription of the microRNAs into cDNA was done with the TaqMan MicroRNA Reverse Transcription Kit (Life Technologies, Thermo Fisher Scientific, Madrid, Spain) and TaqMan microRNA assays specific for miR-21, miR-146a, miR-223, miR-483, and U6 (Applied Biosystems, Thermo Fisher Scientific, Madrid, Spain) according to the manufacturer's recommendations. PCR was performed in 15 *μ*L reaction mixtures using the MJ Mini Personal Thermal Cycler (Bio-Rad Laboratories, S.A., Madrid, Spain). The samples were subjected to thermal cycling parameters of 30 min at 16°C, 30 min at 42°C, and 5 min at 85°C and then kept at 4°C. Real-time PCR reaction was performed in a final volume of 20 *μ*L, containing 1.33 *μ*L of cDNA, 10 *μ*L of TaqMan Universal Master Mix II with no UNG (Life Technologies, Thermo Fisher Scientific, Madrid, Spain), 7.67 *μ*L of RNase-free water, and 1 *μ*L of TaqMan microRNA probe specific for each examined microRNAs (Life Technologies). The cycling conditions were as follows: initial denaturation at 50°C for 2 minutes, followed by enzyme activation at 95°C for 10 minutes, and 40 cycles of 95°C for 15 seconds and 60°C for 1 minute. PCR was performed using an Agilent Technologies Stratagene Mx3005P System (Life Technologies, Thermo Fisher Scientific, Madrid, Spain). All real-time PCR reactions were performed in triplicate. After the reaction, the threshold cycle values were determined using fixed threshold settings. When the average of miRNA expression was ≥35 *C*_*t*_, it was not included in analysis. Data were analyzed using the SDS 2.3 and RQ Manager 1.2 software packages (Life Technologies), and relative gene expression was generated using the 2^−ΔΔCT^ method (Δ*C*_*t*_ target gene − Δ*C*_*t*_ control gene). All miRNAs were calibrated against spike-in control miR-39 (external control). As there is no consensus on endogenous stable miRNAs in the circulation for normalization and quantification of circulating miRNAs, the expression levels were normalized both against U6 snRNA (internal control) and against the average *C*_*t*_ of the control group (general normalization) [21, 22].

### 2.5. Determination of Advanced Oxidation Protein Products

Advanced oxidation protein products (AOPPs) were measured spectrophotometrically on a microplate reader. Samples were calibrated with chloramine-T solution that in the presence of potassium iodide absorb at 340 nm [23]. 200 *μ*L of plasma diluted 1/5 in PBS, 10 *μ*L of PBS, and 20 *μ*L of concentrated acetic acid were added to sample wells. The standard curve was made with 10 *μ*L of 1.16 M potassium iodide, 200 *μ*L of chloramine-T solution (0–100 nmol/mL), and 20 *μ*L acetic acid. The absorbance of the reaction mixture was immediately read at 340 nm on a microplate reader against a blank containing 200 *μ*L PBS, 10 *μ*L potassium iodide, and 20 *μ*L of acetic acid. The AOPP concentration was expressed in nmol/mL of chloramine-T equivalents.

### 2.6. Determination of Lipid Peroxidation

Plasma samples were thawed and centrifuged at 5000*g* x 5 min at 5°C, and 200 *μ*L of the supernatants were used for lipid peroxidation (LPO) measurement. For this purpose, a commercial LPO assay kit that estimates both malondialdehyde and 4-hydroxyalkenals was used (Bioxytech LPO-586 assay kit, Oxis Research, CA, USA). Absorbance was read at 586 nm, and lipid peroxidation concentration was expressed in nmol/mL [24].

### 2.7. Measurement of Plasma Cytokines

The Affymetrix's ProcartaPlex Simplex Kits refs EPX010-10213-901 for IL-6, EPX010-10204-901 for IL-8, EPX010-10215-901 for IL-10, and EPX010-10223-901 for TNF*α* (Labclinics, S.A., Barcelona, Spain) were used to profile expression of these cytokines. The assay was performed according to the manufacturer's instructions. Briefly, 50 *μ*L of working solution containing multiple microbeads labeled with specific antibodies against each cytokine was added into each well, washed twice with 200 *μ*L of wash buffer, and filtered to dryness. Then, 25 *μ*L thawed plasma aliquots diluted 1 : 4 with the specific LINCOplex sample diluent was added to each well and incubated for 60 min at room temperature. After a wash step (twice) with 200 *μ*L wash buffer, the beads were incubated with 25 *μ*L of the detection antibody cocktail containing a specific antibody to each cytokine for 30 min at room temperature. The mixture was washed twice with 200 *μ*L wash buffer; the beads were incubated with 25 *μ*L of the streptavidin-phycoerythrin solution for 30 min at room temperature and washed twice again. The beads were resuspended with 100 *μ*L of buffer and the concentration of each cytokine was determined using the array reader (Bio-Rad Laboratories, Madrid, Spain). A parallel standard curve was constructed for each cytokine. Cytokine levels were expressed in pg/mL.

### 2.8. Statistical Analysis

Data were analyzed using SPSS version 20.0 (WPSS Ltd., Surrey, UK), and graphs were generated using GraphPad Prism 6 scientific software (GraphPad Software Inc., La Jolla, CA, USA). Data were assessed for normal distribution with the Shapiro-Wilk test, and most of them do not have normal distribution. Nonparametric tests (Mann–Whitney rank test) were used to examine any differences among groups. Correlations between 2 parameters were assessed using simple linear regression. Binomial logistic regression analysis was used to explore the association between plasma miRNAs, oxidative biomarker levels, and the presence of frailty. Results are displayed as odds ratios (Exp(*B*)) and 95% confidence intervals (CI). Differences were considered statistically significant at *P* values < 0.05. Data are expressed as means ± standard error of the mean (SEM).

## 3. Results

### 3.1. Characteristics of the Studied Subjects

Table 1 shows the profile of the subjects of the study. The control group was constituted by 22 healthy adults, 15 males, and 7 females, with a mean age of 20.5 ± 2.4 years. Two groups of aged subjects, robust and fragile, aged 76.6 ± 5.3 and 84.4 ± 5.6 years, respectively, were also enrolled in the study. As expected, fragile patients have a lower index of daily physical activity and higher physical dependency than those robust patients. Hypertension was present in a similar extent in both aged groups of subjects.

### 3.2. Plasma miRNA Expression in Studied Samples

**Figure 1:**
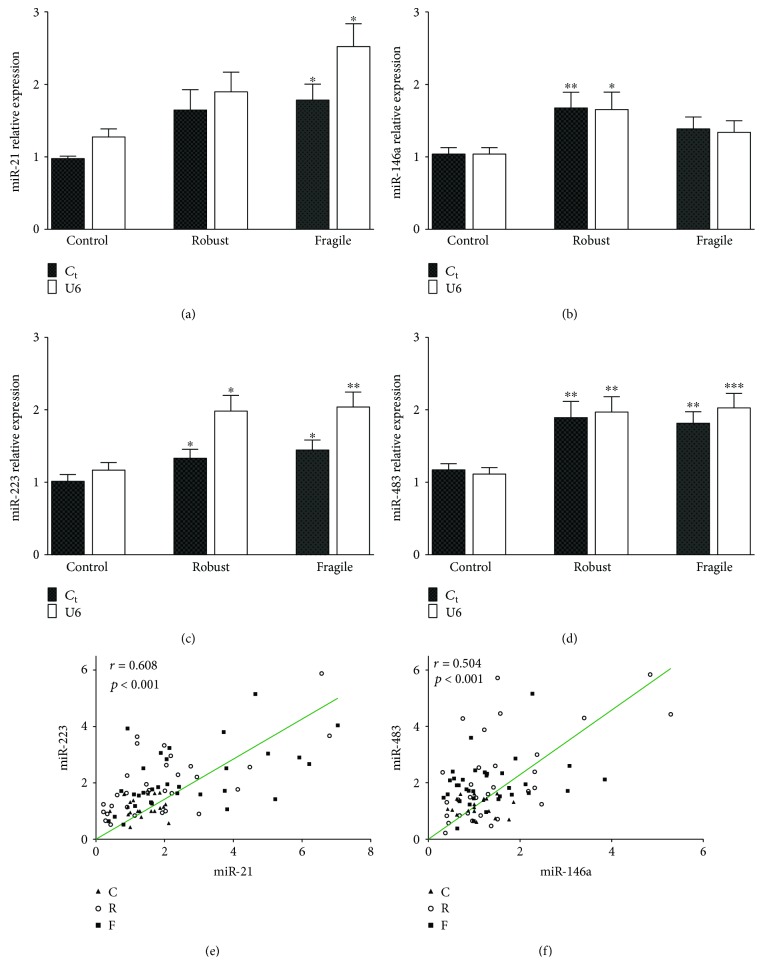
miRNA levels in control and aged groups (a)–(d). Relative expression of these miRNAs was calculated using the 2^−ΔΔCt^ method. The expression levels were normalized against U6 snRNA and against the average of the control group (*C*_*t*_, general normalization). Data are presented as means ± SEM. ^∗^*P* < 0.05, ^∗∗^*P* < 0.01, and ^∗∗∗^*P* < 0.001 versus the control group. Regression and correlation analysis with relative expression of miRNAs, calculated using analysis of the Spearman correlation coefficient. Only those miRNAs that had significant correlation are shown (e) and (f). C: control group; R: robust group; F: fragile group.

The relative cycle threshold (*C*_*t*_) for U6 small nuclear RNA was used as an endogenous control for normalizing the respective miRNAs *C*_*t*_ as previously described [22]. Because some are concerned with the use of this method for circulating miRNAs, we also normalized with the average *C*_*t*_ of the control group. Both normalization methods, however, yield similar results and statistical differences between groups. Compared with the control group, the robust group had higher expression of miR-146a, miR-223, and miR-483, while the fragile group showed higher expression of miR-21, miR-223, and miR-483 (Figures 1(a), 1(d), and 1(d)). So, except for miR-21 in the robust group and miR-146a in the fragile group, age courses with enhanced expression of miRNAs were analyzed (correlation between miR-223 expression and age (*r* = 0.359; *P* < 0.001) and between miR-483 expression and age (*r* = 0.2461; *P* < 0.01) was significant, data not shown). Women had increased expression of miR-483 compared with men (2.12 ± 1.18 versus 1.44 ± 0.99, *P* < 0.001, data not shown). Thus, the results from both methods of normalization guarantee the identification of these miRNAs in our samples, and then, we used normalization for U6. Positive correlations between miR-223 and miR-21 (*r* = 0.608, *P* < 0.001) and between miR-483 and miR-146a (*r* = 0.504, *P* < 0.001) were found (Figures 1(e) and 1(f), respectively).

### 3.3. Markers of Inflammation and Oxidative Stress in Studied Samples

**Figure 2:**
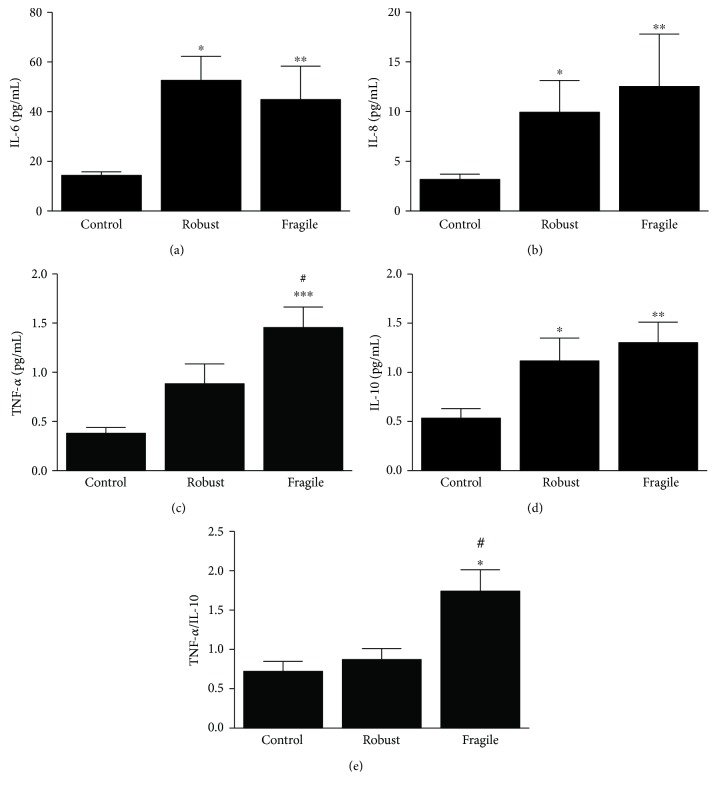
Plasma levels of IL-6, IL-8, TNF*α*, and IL-10 in control and aged groups of subjects ((a)–(d)). TNF*α*/IL-10 ratio in the three studied groups (e). Data are presented as means ± SEM. ^∗^*P* < 0.05, ^∗∗^*P* < 0.01 and ^∗∗∗^*P* < 0.001 versus the control group. ^#^*P* < 0.05 versus the robust group.

**Figure 3:**
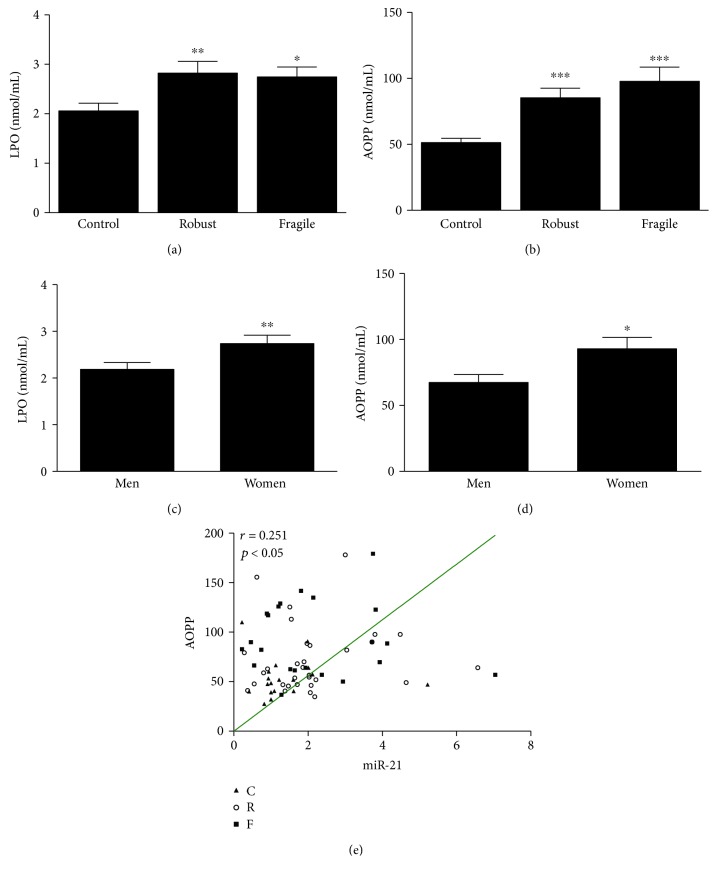
Lipid peroxidation (LPO) and advanced oxidation protein product (AOPP) levels in plasma of subjects of three studied groups ((a) and (b)). LPO and AOPP levels in plasma were also classified according to gender ((c) and (d)). Data are presented as means ± SEM. ^∗^*P* < 0.05, ^∗∗^*P* < 0.01, and ^∗∗∗^*P* < 0.001 versus the control group. Correlation analysis between miR-21 relative expression and AOPP levels (e), calculated using analysis of the Spearman correlation coefficient. C: control group; R: robust group; F: fragile group.

Regarding the inflammatory markers, here measured, we found that IL-6, IL-8, and IL-10 levels increased significantly in both aged groups compared with those in the young control (Figures 2(a)–2(c)), with a trend to display higher levels of IL-8 in the fragile group. In addition, TNF*α* becomes significantly higher in the fragile group compared with that in control and robust ones (Figure 2(c)). Regarding inflammatory/anti-inflammatory balance measured by the index TNF*α*/IL-10, we detected significant difference between fragile subjects and controls, as well as between fragile and robust subjects (Figure 2(e)). No differences between men and women were detected in the levels of the cytokines measured. Because of the connection between miRNAs and inflammation, we next studied the existence of any correlation between these miRNAs and proinflammatory cytokines. Our data show only positive correlation between miR-483 and IL-8 (*r* = 0.430, *P* < 0.05) (data not shown). As expected, age was accompanied with increased oxidative stress, which is reflected in significantly higher levels of LPO and AOPP in plasma of all aged patients compared with youngers (Figures 3(a) and 3(b)). No significant differences were found between aged robust and fragile groups. Gender comparison of plasma levels of LPO and AOPP reported that women have significantly higher levels of these biomarkers than men (Figures 3(c) and 3(d)). Additionally, we found that miR-21 and AOPP correlated significantly (*r* = 0.251, *P* < 0.05) (Figure 3(e)). Logistic regression analysis suggested that the expression of miR-21 and index TNF*α*/IL-10 correlates positively with frailty (Exp(*B*) = 1.423, 95% CI: 1.079–1.878, *P* = 0.013 for miR-21 and Exp(*B*) = 2.433, 95% CI: 1.019–5.810, *P* = 0.045 for TNF*α*/IL-10).

## 4. Discussion

We present data that support that oxidative stress and cytokines, as well as subclinical inflammation, measured through miRs (miR-223 and miR-483), increase with age. Also, miR-21 become significantly higher in the group of fragile patients. We show here the association between miR-21 with frailty and AOPP and between miR-483 with frailty and IL-8; moreover, index TNF*α*/IL-10 was significantly elevated in the fragile group. These results point to the utility and feasibility of the determination of miRNAs together with parameters of inflammation and oxidative stress in a routine blood analysis to detect early signs of frailty during aging.

It has been suggested that low miR-21 expression is related with a healthier aging [25]. Different mechanisms of action of miR-21 in age have been reported. Circulating miR-21 can bind to toll-like receptors (TLRs) in surrounding immune cells, leading to NF-*κ*B pathway activation and increased secretion of proinflammatory cytokines including IL-6 and TNF*α* [26]. Moreover, miR-21 directly modulates TGF-*β* signaling by targeting the TGF-*β*R2 in different cellular models. This signaling pathway can interconnect inflammation, senescence, and cancer [27]. The increase in serum miR-21 in response to an acute exhaustive exercise in congestive heart failure patients further supports its relationship also with muscle condition and, thus, sarcopenia [28]. Here, we report a significant increase in plasma levels of miR-21with frailty, supporting its relation with inflammaging and age-related diseases.

Regarding miR-146a and unlike miR-21, the former increase with age but not with frailty. miR-146a/b is a component of a negative feedback loop that downregulates the levels of IRAK1 by repressing TNF receptor-associated factor 6 (TRAF6) and interleukin-1 receptor-associated kinase 1 (IRAK1) expression [29]. An upregulation of miR-146a in cells with high senescence-associated secretory phenotype (SASP) leads to increase IL-1*α* levels enhancing IRAK1 and NF-*κ*B-dependent production of IL-6, IL-8, and also miR-146a/b [30]. Hence, miR-146a can prevent an excessive production of inflammatory mediators, thus limiting some of the potentially deleterious effect of SASP [30]. Our results showed that miR-146a was lower in fragile subjects that in robust aged subjects, which may trigger the proinflammatory pathway. In fact, fragile patients tend to have higher levels of IL-8 and high levels of TNF*α* than healthy aged subjects.

Initially described as a key modulator of hematopoietic lineage differentiation, miR-223 is one of the most abundant miRNAs in plasma. miR-223 regulates the pathways related with immune responses by affecting different targets; these include insulin-like growth factor 1 receptor (IGF-R1), IKK*α*, STAT3, FOXO1, NLRP3, and Roquin, and it may induce the activation of NF-*κ*B in macrophages during their differentiation [29]. Circulating miR-223 has been positively associated with inflammation and fibrosis muscle-deficient mice [31]. Our results shown that miR-223 expression increased in both groups of aged patients, and thus, miR-223 could be considered a biomarker of aging but not of frailty. This assumption coincides with recent investigation of the epigenetic network involved in human aging [32]. Nevertheless, newest findings suggest that miRNAs do not act individually but rather exert a cooperative control over different signaling pathways. Here, we found a positive correlation between miR-21 and miR-223. Whereas both of them are related with aging, the former is also related with frailty and AOPP. Thus, miR-21 could be used to detect a risk of frailty in the patients.

With regard to miR-483, different targets have been described. Clokie et al. demonstrated that miR-483 extracted from the rat pineal gland act as suppressor of *arylalkylamine-N-acetyltransferase (aanat)* mRNA expression, the key enzyme in melatonin synthesis [33]. High levels of miR-483 suppress N-acetylserotonin production, the melatonin precursor, leading to a reduction in melatonin synthesis. Our data showed that miR-483 correlated with IL-8 and its expression levels significantly increased with age. Since melatonin production decays with age, the increased expression of miR-483 in aged patients could underlie the age-dependent melatonin decay. Nevertheless, this affirmation requires further confirmation because we did not measure the levels of melatonin in the groups of subjects here studied.

Human aging can be considered as a complex process that combines inflammation and oxidative stress. In our study, we found a significant increase in the levels of the proinflammatory cytokines IL-6, IL-8, and TNF*α* with age, Moreover, some studies proved that elevated levels of TNF*α* can increase muscle catabolism by suppressing the Akt/mTOR pathway [34]. The tendency to increase IL-6, IL-8, and significantly high levels of TNF*α* in the group with frailty here reported, as well as increased inflammatory/anti-inflammatory balance measured by the index TNF*α*/IL-10, suggests a role for these cytokines in muscle deterioration. Together with inflammation, we report here the increase in LPO and AOPP levels, reflecting the lipid and protein oxidative damage. Again, these ROS did not show differences with frailty, although women have significantly higher levels of LPO and AOPP than men.

The use of different animal models for the study of changes in aged muscle confirms that circulating miRNAs indicate that while muscle and serum have distinct age-related changes in miRNA expression, some miRNAs showed the same directionality in expression changes [35]. Together, our data suggest a relationship between c-miRNAs and aged patients with and without frailty. Elevated levels of miR-21 in frailty indicate an activation of innate immunity associated with age, and it suggests miR-21 as a biomarker of human muscle frailty. On the other hand, the reduction in miR-146a levels in frailty can promote NF-*κ*B-dependent inflammation. The increased expression of circulating miR-483 in the experimental group may contribute to the decreased production of melatonin due to age. All these changes are accompanied by increased levels of inflammatory and ROS markers, indicating the presence of chronic inflammation associated with age. Women, who have increased AOPP, LPO, and miR-483 compared with men, can be considered as the population most vulnerable to frailty.

## 5. Summary and Conclusions

We confirmed that aging and frailty are companied with higher oxidative and inflammation states, such as increasing expression of c-miR-21, c-miR-223, and c-miR-483, reflecting the changes that occur at the molecular level in the aging process.

Our results point that the miR-21 and TNF*α*/IL-10 ratio may be considered as possible biomarkers for age-related frailty. Enhanced understanding of alterations in c-miRNA expression with aging and in different pathological condition may result in a relevant diagnostic tool and offers more information on mechanisms involved in the aging process. Detection and identification of stable miRNAs in blood has opened a new possibility in systemic biomarker research directed at improving clinical diagnosis and prognosis.

## Figures and Tables

**Table 1:** Characteristics of the studied subjects.

Item	Control group	Aged without frailty	Aged with frailty
Number of subjects	22	34	40
Age	20.5 ± 2.4	76.6 ± 5.3	84.4 ± 5.6
Gender (male/female)			
Male	19 (86%)	12 (35%)	13 (33%)
Female	3 (14%)	22 (65%)	27 (67%)
<80 years old (M/F)	0	24 (10/14)	8 (2/6)
≥80 years old (M/F)	0	10 (2/8)	32 (11/21)
Physical activity	n.m.	79.69 ± 8.05	10.77 ± 2.76^###^
Physical dependence	0%	12%^∗∗∗^	75%^###^
Hypertension	0%	38%^∗∗∗^	32%^∗∗∗^

Age and physical activity are expressed as the means ± SEM. M/F: male/female; n.m.: not measured. ^∗∗∗^*P* < 0.001 versus the control group; ^###^*P* < 0.001 versus aged without frailty.

## Data Availability

The datasets generated during and/or analysed during the current study are available from the corresponding author on reasonable request.
